# Effect of a mix of condense and hydrolysable tannins feed additive on lactating dairy cows’ services per conception and days open

**DOI:** 10.1016/j.vas.2025.100434

**Published:** 2025-02-24

**Authors:** Alejandro R Castillo, Julio A Di Rienzo, Damiano Cavallini

**Affiliations:** a University of California, Cooperative Extension, Merced, CA. 95340, USA; b Facultad Cs. Agropecuarias, Universidad Nacional de Córdoba. 5000, Argentina; c Dipartimento di Scienze Mediche Veterinarie, Alma Mater Studiorum University of Bologna, Bologna, Italy

**Keywords:** Dairy cows, Condensed and hydrolysable tannins, Services per conception, Days open

## Abstract

A commercial dairy farm with two dairy units (about 3000 cows each), similar management and diets was used to evaluate the effect of a feed additive based on a mix of condense and hydrolysable tannins (ByPro®) on lactating cow services per conception and days open. The experiment was divided in three evaluation periods. Period I, January-March (without-feed additive), Period II, April-September, all cows in one dairy unit received the mix-tannins feed additive at 0.3 % of the total estimated DMI per cow. Period III, October-December (without-feed additive). Services per conception was modeled as a Poisson count using a generalized linear model with log link function. Days open was modeled with a generalized linear model for a gamma variable with log link function. The models included: dairy units (2), periods (3), and their interactions. No significant differences were observed in Period I. During the evaluation Period II, the mix tannins feed additive increased the artificial insemination efficiency, reducing (*P* < 0.01) the number of services of conception (- 9 %), from 2.20 to 2.02 and days open (*P* < 0.01) from 92.5 to 87 days (- 6.3 %). Comparable significant effects were observed in Period III without the mix-tannins feed additive that might be related to possible rumen microbiota carried over effects of the cows consuming the mix-tannins for 6 months. Finally, the treatment improved performances on the conception rate and pregnancy rate. The mix of condensed and hydrolysable tannins feed additive (ByPro®) improved reproduction efficiency in lactating cows, by reducing services per conception number and days open.

## Introduction

Tannins are defined as complex polyphenolic compounds produced by plants ranging in a molecular weight from 500 to 3000 Daltons and have broadly been defined as either condensed or hydrolysable based on their chemical structure (Marshall et al., 2022).

In ruminants, different studies show that tannins play an important role improving dietary energy and nitrogen utilization efficiency. The ability of tannins to bind proteins have been well described. Tannins modify the ruminal fermentation by formation of poorly degradable complexes with proteins that are stable within the pH range of 3.5 to 7.0, and also by reducing quantity of immediately degradable fractions. Such formed complexes, protect proteins from microbial hydrolysis and deamination in the rumen, and increased the quantity of proteins that are degraded in abomasum and small intestine (Davidović et al., 2019). Tannins that bind to dietary protein increase the N flux from the rumen to the small intestine, this process was referred to as “ruminal escape protein” (Muller-Harvey, 2006).

Tannins are known to have protein binding properties and therefore will reduce the available protein in the rumen and improve its utilization (Marshall et al., 2022). Results of other studies mixing condensed and hydrolysable tannins with high performance lactating dairy cows were very consistent (Aguerre et al., 2015, 2016). Moreover, feeding a mix of quebracho and chestnut tannin extract are adequate to reduce excretion of environmental labile urinary N and increase N utilization efficiency by improving true protein content in milk, and reducing milk urea-N and N-excretion (Marshall et al., 2022). These secondary plant metabolites have also a property to form reversible and irreversible complexes with polysaccharides (cellulose, hemicelluloses, starch and pectin), alkaloids, nucleic acids and minerals (Davidović et al., 2019).

Recent research highlights the potential of phytogenic dietary additives, particularly tannins, to enhance animal productivity through their multifaceted effects. Tannins not only influence the digestive process by binding dietary proteins, thereby improving nutrient utilization, but they also exert significant indirect effects on gastrointestinal microbiota (Diaz Carrasco et al., 2017).

All these studies indicates that tannins may increase directly or indirectly the efficiency of energy utilization. Lucy *et al*. (2013) concluded that the inability of the early postpartum cow to achieve an adequate entry rate for glucose relative to whole-body demand is a possible mechanism that links postpartum physiology and nutrition to reproduction in dairy cows.

Functional and novel feed additives, such as tannins, are gaining attention in animal nutrition for their potential to enhance productivity and health. These additives are designed to improve the efficiency of nutrient utilization, promote gut health, and modulate the microbiota, leading to better overall animal performance. Tannins, in particular, have shown promise not only in binding dietary proteins to improve nutrient absorption but also in exerting positive effects on the gastrointestinal microbiome. Their multifaceted roles in animal diets highlight the need for further exploration of these additives as valuable tools for improving livestock productivity and sustainability in modern agriculture (Vastolo et al., 2024; Gasparini et al., 2024).

The hypothesis of this research is that mixing condensed and hydrolysable tannins supplemented to high producing lactating dairy cows should improve reproductive efficiency by reducing services per conception and open days. Long-term studies feeding a mix of tannins to evaluate reproduction efficiency variables in dairy cows were not found. The aim of this on-farm research was to evaluate the long-term effect of a commercial mix of condensed and hydrolysable tannins feed additive (ByPro®) on lactating dairy cows services per conception and days open.

## Materials and methods

The trial was conducted at a dairy farm in Santa Fe Province, Argentina, from January to December, involving two split free-stall units housing over 3000 Holstein cows each. Both units were managed under similar practices and diets, which included a Total Mixed Ration (TMR) designed to meet the nutritional needs of lactating cows.

Environmental conditions at the farm, including temperature, humidity, and seasonal variations, play a crucial role in influencing dairy cattle health and productivity. Santa Fe Province experiences a temperate climate with distinct seasons, where summer temperatures can reach up to 35 °C (95°F) and winter temperatures can drop to around 5 °C (41°F). High temperatures can cause significant heat stress in cows, leading to reduced feed intake, decreased milk production, and impaired reproductive performance. To counteract these negative effects, various management strategies were implemented. These included providing ample shade to minimize direct sun exposure, ensuring proper ventilation to enhance air circulation and cooling, and maintaining continuous access to fresh, clean water to support hydration and thermoregulation. Additionally, some farms introduced cooling systems such as fans and sprinklers to further alleviate heat stress and improve overall animal welfare and productivity. All these measures are reported to be effective in reducing heat stress effects (Felini et al., 2024)

Moreover, the farm utilized standard protocols for cow handling and health monitoring, which included regular veterinary checks, vaccination schedules, and nutritional assessments to ensure optimal animal health and welfare. The consistent management practices and environmental monitoring were critical for maintaining a stable experimental environment and ensuring the replicability of the results.

All lactating cows in one dairy unit received a feed additive based on a mix of condensed and hydrolysable tannins (ByPro®) and the other dairy unit was used as a Control. The experiment was divided in three evaluation periods (without, with, and without feed additive). Period I, from January to March (without-feed additive), Period II, from April to September, cows received mix of condense and hydrolysable tannins feed additive ByPro® at 0.3 % of the total estimated DMI per cow. The total tannin content was checked by the producers and certified during the trial. Period III, October to December (without-feed additive).

All lactating cows in both dairy units received the same dietary ingredients. TMR diets were based on: 7 % Alfalfa hay, 26.4 % Corn silage, 5 % Wheat silage, 4 % Alfalfa silage, 5.8 % Corn grain, high moisture, 14.2 %. Corn grain, ground dry, 20 % Corn gluten feed, 7.2 % Soybean meal, 5.8 % Soybean grain, 1.1 % Fat, bypass, and 3.5 % Mineral and Vitamin Premix. The dietary difference between Control and ByPro® dairy units was the mix of tannins included in the TMR premix of ByPro® dairy cows. The DMI was estimated based on the total daily mixed ration supplied divided by the number of lactating cows and adjusted for DM contents. The DM content in the TMR were calculated based on the DM content of each dietary ingredient. All feed components were sampled and analyzed before the start of the trial. The diet was formulated based on CNCPS guidelines. Daily TMR offer in each group was estimated to maintain about 3, 5, and 2 % refusal in fresh, mid and late lactation cow groups, respectively. The DMI intake and refusal were adjusted weekly.

The two dairy units have similar cows’ management and handled by the same insemination team, which used same standard protocols for artificial insemination and records of services per conception and days open for both dairy units.

To characterized TMR nutrient contents, along the trial TMR samples were taken from the whole feed bank at the high production cows’ pen in both dairy units and analyzed for CP, ADF, NDF, Fat, Ash, Ca, P, Mg, Cl, K, Na, Cu, Fe, Mn, and Zn, by standard methodologies (AOAC, 1990, 1999) as reported in previous studies (Cavallini et al., 2018; Palmonari et al., 2021; Koakoski et al., 2024). Results are reported in Table 1.

**Table 1:** TMR ingredients composition, estimated dry matter intake (DMI), milk yield/cow, and dietary nutrient content.

*item*	ByPro Dairy	Control Dairy
TMR ingredients ^(1)^		
Alfalfa hay, %	7.0	
Corn silage, %	26.4	
Wheat silage, %	5.0	
Alfalfa silage, %	4.0	
Corn grain, high moisture, %	5.8	
Corn grain, ground dry, %	14.2	
Corn gluten feed, %	20.0	
Soybean meal, %	7.2	
Soybean grain, %	5.8	
Fat, bypass, %	1.1	
Premix, **%**	3.5	
Nutrient contents		
CP, %	17.40	17.75
NDF, %	27.81	27.20
ADF, %	19.10	18.00
Fat, %	5.10	5.10
Ash, %	8.84	8.39
Ca, %	0.99	0.82
P, %	0.42	0.47
Mg, %	0.37	0.33
K, %	1.43	1.35
Na, %	0.20	0.19
Cl, %	0.31	0.28
Cu, ppm	16.50	14.50
Mn, ppm	53.25	44.92
Zn, ppm	62.42	57.25

1TMR ByPro Dairy includes the mix of tannins in the Premix.

### Statistical analysis

The two dairy units were compared through 2015 on three periods: (I) without (Jan. to March), (II) with (Apr. to Sept.), and (III) without the feed additive ByPro® (Oct. to Dec.), respectively. In period II, all lactating cows in one unit received the feed additive ByPro®, the other unit was used as Control. Variables evaluated were services per conception and days open.

Services per conception was modeled as a Poisson count using a generalized linear model with log link function. Days open is a waiting time variable and have an asymmetric distribution. It was modeled with a generalized linear model for a gamma variable with log link function. The use of GLMs was justified as services per conception follows a Poisson distribution (count data), while days open follows a gamma distribution (waiting-time data), ensuring appropriate modeling of non-normal and skewed distributions.

The models for services per conception and days open included: dairy unit (2), periods (3), and their interaction as fixed effects. The models were fitted using glm function of R (Pollesel et al., 2020), and the interfaced InfoStat statistical software (Di Rienzo *et al*., 2017).

## Results

Estimated daily DMI and milk yield per cow through the trial were similar in both dairy farms averaging 25.6 kg and over 36 kg of milk, respectively.

Results are presented in Table 2. >4000 milking cows were evaluated for services per conception and days open, about 2000 cows in each dairy unit. No significant differences were observed from January to March (Period I) without the feed additive. During the evaluation Period II from April to September, the mix tannins feed additive increased artificial insemination efficiency *(P* < 0.01) reducing the number of services of conception in almost - 9 %, from 2.20 to 2.02, which can represent a cost reduction in semen, labor, and calving interval. ByPro® also affected days open (*P* *<* 0.01), they were reduced from 92.5 to 87 days (−6.3 %).

**Table 2:** Effect of a mix of tannins feed additive ByPro® on lactating dairy cows services per conception and days open.

	Cows evaluated (n)		Services per conception			Days open		
Evaluation periods ^(1)^	ByPro®	Control	ByPro® ± SE ^(2)^	Control ± SE ^(2)^	*P*< ^(3)^	ByPro® ± SE ^(2)^	Control ± SE ^(2)^	*P*< ^(3)^
I. Jan. to March	616	608	2.55 ±0.06	2.63 ±0.07	Ns	102.6 ± 1.95	106.7 ± 2.04	ns
II. April to Sept.	1114	1173	2.02 ±0.04	2.20 ±0.04	*	87.0 ±1.23	92.5 ±1.27	*
III. Oct. to Dec.	257	255	1.83 ±0.08	2.19 ±0.09	*	78.9 ±2.32	91.3 ±2.70	*
Total cows	1987	2036						

1 Period I. Jan. toMarch: no supplementation; Period II. April to Sept.: ByPro cows received 0.3 % of DMI feed additive mix tannins in ByPro® dairy. Period III. Oct. to Dec. no supplementation.

2 SE: standard errors of the means.

3 ns: non-significant, * *P* < 0.01.

In Period III (October to December), after the feed additive was removed, reproductive performance continued to improve. Services per conception decreased by nearly 20 % (*P* *<* *0.01*) in the ByPro® group compared to the control, dropping from 2.19 to 1.83, while days open were reduced by 15.6 %, from 91.3 to 78.9 days (*P* *<* *0.01*). The continued improvements in reproductive parameters suggest a possible carry-over effect, potentially linked to persistent changes in rumen microbiota and enhanced metabolic efficiency.

**Fig. 1:**
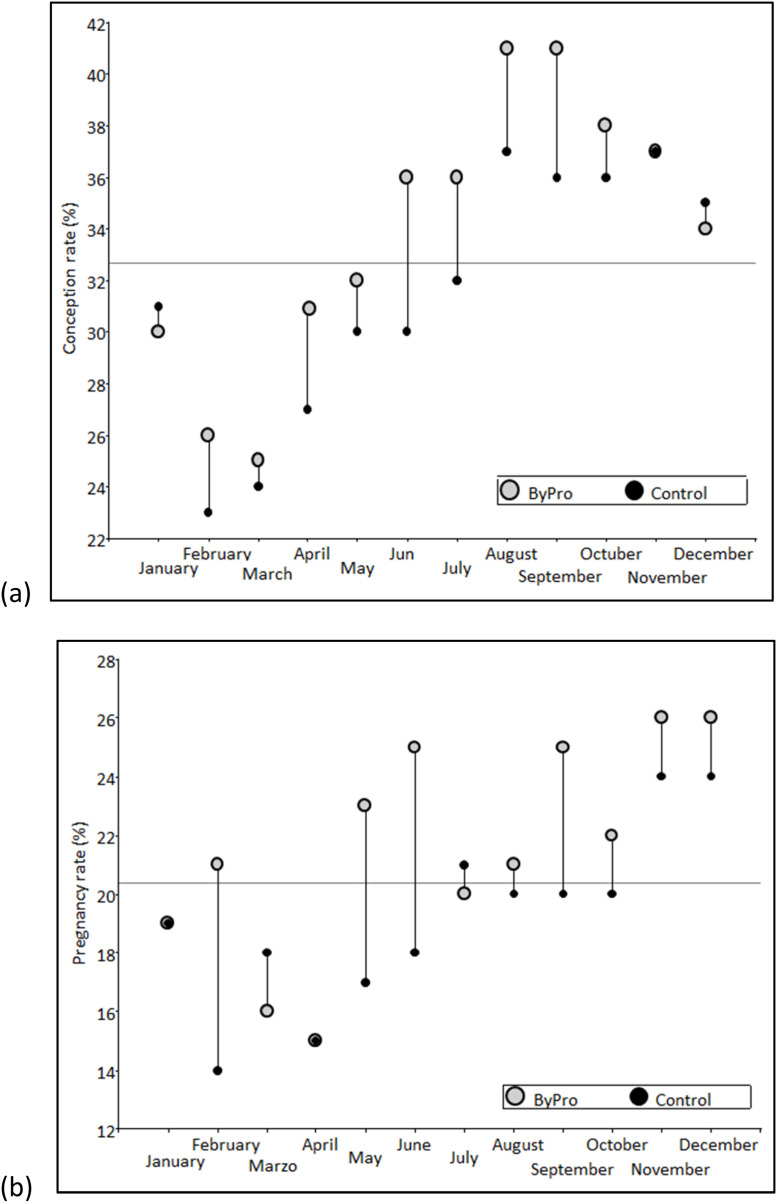
Conception rate (a) and pregnancy rate (b) trends in lactating dairy cows consuming a mix of tannins (ByPro® Dairy) from April to September, respect to the Control Dairy.

Fig. 1a shows the conception rate trends in lactating dairy cows supplemented with ByPro® compared to the control group during the supplementation period (April to September). The ByPro® group consistently exhibited a higher conception rate throughout the evaluation period, indicating that cows receiving the tannin supplement required fewer artificial inseminations to conceive. This suggests improved reproductive efficiency, likely due to enhanced nutrient utilization and metabolic balance.

Fig. 1b presents the pregnancy rate trends in both groups over the same period. The pregnancy rate was consistently higher in the ByPro® group compared to the control, demonstrating that cows supplemented with tannins not only conceived more efficiently but also had a greater likelihood of maintaining pregnancy. This effect may be attributed to improved energy balance, reduced oxidative stress, and potential positive impacts on reproductive physiology.

## Discussion

The aim of this study was to evaluate the long-term effects of a commercial mix of condensed and hydrolysable tannins (ByPro®) on reproductive efficiency in lactating dairy cows, specifically by assessing its impact on services per conception and days open. The study was conducted on a commercial dairy farm, comparing two dairy units under similar management conditions across three periods: before supplementation, during supplementation, and after supplementation withdrawal.

In commercial dairy farms, the general practice is to breed cows early, to obtain a calving interval of 12 to 13 months. Calving intervals and days open are correlated. Early studies (Louca & Legates, 1968) analyzed dairy production losses due to days open. They estimated an average decreased of 2.4 ± 1.09 kg of milk and 0.112±0.040 kg of fat for each additional day open. For second and third lactations cows, there was a reduction of 3.58 and 3.68 kg of milk, respectively. A positive response was estimated for primiparous cows, increasing 1.16 kg of milk for each additional day open, which was attributed to a higher persistency in first lactation animals. It is suggested a calving interval of 13 month for primiparous and 12 months for multiparous cows as an optimal length for attaining maximum milk production. In fact, using records of primiparous (6350 lactations) and multiparous cows (almost 18,000 lactations) from a commercial dairy farms Olds *et al*. (1979), analyzed the effects of days open on the economic aspects of current lactations. Within herds each open day between 40 and 140 days of lactation resulted in an average 4.5 kg less annual milk during current lactations of first-calf heifers and 8.6 kg less for cows in later lactations. The differences between these results with the previous of Louca and Legates (1968) could be related to the length of days open or calving interval included in the analysis.

The concept of longer open days or calving intervals and its effects on milk production has been discussed in different studies. For example, Oltenacu et al. (1980) stated that the mechanisms by which pregnancy adversely affects milk production are not yet fully understood. However, they concluded that these effects are primarily due to hormonal control of milk secretion and the partitioning of nutrients for various biological functions. In the same study, Oltenacu *et al*. confirmed that the inhibitory effects of pregnancy on milk yield largely result from redirecting available energy from milk production to pregnancy requirements and replenishing energy reserves for the next lactation.

Arbel at al. (2001), demonstrated an advantage for longer days open for milk production and profitability. They examined whether extending calving interval in high yielding dairy cows would affect profitability during the current and subsequent lactation. Concluding that a delay of 60 days with respect to the voluntary waiting period in the beginning of the inseminations of high yielding cows has economic advantages and allows the farmers an option for decisions regarding individual cows, and in primiparous cows, because the high persistence of their milk production, which agrees to the early observation of Louca and Legates (1968).

Typically, the voluntary waiting period for dairy cows (the postpartum period when cows are not bred, even if estrus occurs) lasts around 40–50 days. Arbel et al. (2001) suggested extending this by an additional 60 days, resulting in a possible waiting period of 100–110 days. In our trial, open days were from almost 80 to 107 days. Particularly, open days during the supplementation period (II) with the mix-tannins averaged 90 days. Any improvement lower than 100–110 days open should be beneficial for the cows. In our research, this benefit represented a reduction of 5.5 days open for the cows supplemented with ByPro® respect to the Control lactating cows, with an additional saving in semen, labor and feed, and supporting the hypothesis that ByPro tannins might be positive for improving cows’ reproduction performance.

The significant effects observed in Period III (October to December) without the tannin feed additive may be due to carry-over effects on the rumen microbiota from the cows consuming tannins for six months during Period II (April to September). Molecular rumen microbiota studies have identified a “core microbiome” dominated by phyla Firmicutes and Bacteroidetes (Weimer, 2015). Min et al. (2014) investigated the changes in ruminal bacteria diversity and animal performance in goats to plants tannins. They used quebracho and chestnut tannins and both significantly affected the proportions of the most dominated rumen phyla, Firmicutes and Bacteroidetes. Researchers concluded that tannins impact to very specific members of the microbial rumen population, and there is also possible adaptation of ruminal microbiota to tannins with beneficial effects of some class of rumen bacteria. These results align with an eminent review on rumen microbiota by Weimer (2015). The review concluded that the rumen microbial community exhibits both redundancy—overlap of functions among multiple species—and resilience, the ability to recover from perturbation. Together, these properties provide remarkable stability, maintaining digestive function across diverse feeding and management conditions. These properties could be related to rumen microbiota carry over effects of the cows consuming mix-tannins (Period II) on the following Period (III) in this experiment, which were a reduction of - 15.6 % of days open and almost - 20 % services per conception (Table 2). Although the carry-over effects observed in Period III suggest a potential impact on rumen microbiota, without specific microbiome analysis, it is difficult to conclusively attribute these effects to the tannins alone. The "carry-over" effects observed in Period III, where cows that had previously received ByPro® tannin supplementation continued to show improved reproductive efficiency, may be attributed to significant changes in the rumen microbiome. Research indicates that dietary interventions can lead to lasting alterations in microbial populations, potentially enhancing the resilience and adaptability of the rumen ecosystem. For instance, tannins can selectively promote beneficial bacteria, such as Ruminococcaceae, while suppressing less desirable species, thereby reshaping the microbial community structure. This shift can enhance the overall stability of the rumen environment, leading to improved digestion and nutrient absorption even after the cessation of supplementation. Studies have shown that a resilient microbiome can better withstand perturbations, ensuring consistent performance in terms of nutrient utilization and health outcomes. Thus, the observed carry-over effects in reproductive performance may be linked to the enduring benefits of an optimized rumen microbiota, highlighting the importance of considering long-term microbiome dynamics when evaluating the efficacy of feed additives like the one tested in this trial.

Fig. 1 presents two key reproductive metrics: conception rate and pregnancy rate, comparing the group of lactating dairy cows supplemented with the tannin additive to a control group during the supplementation period (April to September). The ByPro® group shows a consistently higher conception rate compared to the control group throughout the trial period (Fig. 1a). This indicates that cows receiving the tannin supplement were more successful in conceiving with fewer artificial inseminations. The improvement in conception rate can be attributed to the potential positive effects of tannins on reproductive physiology, such as enhanced energy and nitrogen utilization, which are crucial for reproductive efficiency in dairy cows. By improving the cows' overall nutritional status, the tannins likely support better estrus synchronization and fertility. The pregnancy rate in the ByPro® group also surpasses that of the control group across the same period (Fig. 1b). The higher pregnancy rate suggests that, beyond conception, the cows supplemented with tannins had a greater chance of sustaining pregnancy. This effect might be linked to the stabilizing impact of tannins on rumen microbiota, which can influence overall health, metabolic function, and reproductive performance. Additionally, improved protein utilization and energy balance, facilitated by tannins, may help cows maintain pregnancies more effectively. Supplementation of tannins extracts has also been reported to exhibit health beneficial effects, such as immunomodulation, anti-inflammatory, antioxidant, etc. (Wang et al., 2024). Early-lactating cows are susceptible to experiencing oxidative stress and have low antioxidant defense (Mammi et al., 2020) leading to a high incidence of pathologies (Cavallini et al., 2022; Magro et al., 2024) and reducing the rumination time (Lamanna et al., 2025). The antioxidant effects of tannins extracts help cows resist oxidative stress. Antioxidant status is closely associated with milk production and reproduction performances, as better antioxidant status allows more energy and nutrients to be allocated toward milk synthesis and oocyte cycle rather than the body's anti-stress processes (Wang et al., 2024).

The reduction in services per conception, as observed in the ByPro®-supplemented group, has both significant economic and welfare implications. Fewer services per conception directly translate into reduced labor costs, as less time and resources are needed for artificial insemination procedures. This leads to cost savings in semen, veterinary care, and labor, making the dairy operation more efficient. Moreover, improving conception rates can shorten calving intervals, resulting in higher milk production and better herd management. From an animal welfare perspective, cows that conceive more easily experience less reproductive stress, contributing to better overall health and productivity. This, in turn, supports improved lactation performance and longevity in the herd, promoting a more sustainable and profitable dairy farming system (Mammi et al., 2020; Buonaiuto et al., 2022, 2023) reducing the amount of the young stock (Ferlizza et al., 2020).

While the study provides strong evidence that ByPro® tannin supplementation improved reproductive efficiency in dairy cows, certain strengths and limitations should be acknowledged. A key strength of this research is its on-farm evaluation under real commercial conditions, enhancing the practical relevance of the findings. The study design, which included a control group and multiple evaluation periods, allowed for a comprehensive assessment of the effects of tannin supplementation and potential carry-over effects. Additionally, the large sample size of over 6000 cows strengthens the reliability of the results.

However, some limitations should be considered when interpreting the findings. Although management practices, environmental conditions, feed quality, cow genetics, and health monitoring were standardized across both dairy units, unaccounted factors such as individual cow variability, undetected health issues, or subtle differences in farm routines could have influenced conception and pregnancy rates. The study relied on routine farm records for reproductive data, which, while practical, may introduce some degree of measurement error or reporting bias. Furthermore, while the one-year duration is relatively long, it remains uncertain whether the observed benefits would persist over multiple lactation cycles or if cows might develop tolerance to the tannin supplement over time.

Another important limitation is the lack of replication across multiple farms, which may restrict the broader applicability of the results. Farm-specific factors such as diet composition, herd management, and environmental stressors could influence how tannins affect reproductive performance. Additionally, while the study suggests a possible carry-over effect linked to rumen microbiota adaptation, microbiome analysis was not performed, leaving the underlying mechanisms speculative.

Further research, including multi-farm trials, extended monitoring across lactations, and detailed microbiome studies, is needed to isolate the effects of ByPro® from other influencing factors, validate its long-term benefits, and better understand its mode of action in improving reproductive performance. Additionally, effectively disseminating these findings through modern communication platforms, such as social media, is crucial for combating misinformation in animal science. Leveraging digital channels like Twitter, Facebook, and Instagram can help bridge the gap between scientific research and public understanding, ensuring that accurate information reaches a wider audience beyond academia. In a parallel vein, a study utilizing Instagram (Lamanna et al., 2024) highlights the effectiveness of social media in conveying complex topics to diverse audiences, demonstrating how digital platforms can enhance public engagement and awareness in specialized fields. Integrating such communication strategies will not only strengthen the impact of this research but also contribute to a more informed and scientifically literate society.

## Conclusions

The mix of condensed and hydrolysable tannins feed additive (ByPro®) improved reproduction efficiency in lactating cows, by increasing artificial insemination efficiency by reducing services per conception (−9 %) and days open (−6.3 %). The mix tannins were supplied for 6 months. Apparently, carry-over effects related to microbiota changes might be affecting the results in the last Period (III) without the mix tannins feed additive in the diet, reducing - 15.6 % of days open and almost - 20 % services per conception. These results open new possibilities to study the long-term effects of mix tannins on dairy cows’ nutrition and reproduction efficiency.

## Ethical statement

The experiment was conducted on a commercial dairy herd in Argentina. No experimental procedures were performed on the animals; instead, we utilized routine data commonly recorded on the farm. All procedures complied with national laws governing animal welfare and veterinary practices; therefore, ethical committee authorization was not required.

## CRediT authorship contribution statement

**Alejandro R Castillo:** Writing – review & editing, Supervision, Resources, Project administration, Methodology, Investigation, Conceptualization. **Julio A Di Rienzo:** Methodology, Investigation, Data curation. **Damiano Cavallini:** Writing – review & editing, Writing – original draft, Data curation.

## Declaration of competing interest

The authors declare no conflict of interest.
